# Influence of primer & probe chemistry and amplification target on reverse transcription digital PCR quantification of viral RNA

**DOI:** 10.1016/j.bdq.2016.08.003

**Published:** 2016-08-27

**Authors:** Fran Van Heuverswyn, Maria Karczmarczyk, Heinz Schimmel, Stefanie Trapmann, Hendrik Emons

**Affiliations:** European Commission, Joint Research Centre, Retieseweg 111, B-2440 Geel, Belgium

**Keywords:** Digital PCR, Reference material, Molecular methods, Molecular probes, RNA virus, Quantification

## Abstract

Compared to other PCR technologies, digital PCR is a potentially highly accurate approach for the quantification of nucleic acid fragments. This study describes the impact of four experimental factors, namely primer and probe chemistry, PCR amplification target, duplexing, and template type, on the measurement results obtained by reverse transcription digital PCR (RT-dPCR) of viral RNA using *influenza A* virus as a model. Along conventional dual labelled probes (DLP), alternative primer and probe chemistries, including Zip Nucleic Acids (ZNAs), Locked Nucleic Acids (LNAs), and Scorpions^®^, were compared with two RNA template types: i) total genomic RNA extracted from cell cultured influenza A and ii) a synthetically prepared RNA transcript (*In vitro* transcribed RNA).

While apparently duplexing or a different PCR target choice did not have a significant influence on the estimated RNA copy numbers, the impact of the choice of primer and probe chemistry and template type differed significantly for some methods. The combined standard uncertainty of the dPCR analysis results has been assessed, taking into account both the repeatability and the intermediate precision of the procedure.

Our data highlight the importance of dPCR method optimisation and the advantage of using a more sophisticated primer and probe chemistry, which turned out to be dependent on the template type. Considerations are provided with respect to the molecular diagnostics of viral RNA pathogens, and more specifically, for precise quantification of RNA, which is of tremendous importance for the development of RNA calibration materials and the qualification of these calibrants as certified reference materials.

## 1 Introduction

Digital PCR (dPCR) builds on the traditional PCR amplification and fluorescence-probe based detection methods, as known from quantitative real-time PCR (qPCR). Therefore, dPCR uses the same primers and probes as the widely applied qPCR, but has been reported to be capable of higher sensitivity and precision due to the underlying principle of limiting dilution, whereby the sample is diluted and partitioned into many separate reaction partitions (chamber-based dPCR) or droplets (droplet-based dPCR) before amplification [1]. Due to another characteristic of dPCR, namely its independence from calibrants containing the DNA or RNA template subject to analysis, the method has become an attractive option for nucleic acid quantification. It has been successfully applied in the certification of reference materials used for standardising qPCR assays widely employed in clinical diagnostics and research areas [2], [3], [4]. Despite these major advantages, several studies have also reported significant bias when measuring both DNA and/or RNA using dPCR [5], [6], [7]. Such discrepancies have been attributed to the choice of the dPCR format (chamber vs droplet-based dPCR), the complexity of the nucleic acid template, detection reagents used, and pre-analytical steps such as nucleic acid extraction [3], [4]. RNA poses a particular measurement challenge due to its instability and the additional step required for detection and quantification: reverse transcription (RT), during which complementary DNA (cDNA) is synthesised.

Another influencing factor could be the choice of the primer and probe system. Conventional dual labelled probes (DLP) with a fluorophore and a quencher, such as a Taqman^®^ probe, are widely employed for (RT-) qPCR and (RT-) dPCR. In recent years, an increasing number of alternative PCR chemistries have become available. These include Locked Nucleic Acids (LNA), Zip Nucleic Acids (ZNA), and Scorpions^®^, among others. Several studies demonstrated that the use of alternative primer and/or probe chemistries may offer improved assay sensitivity [8], [9], [10]. So far, such options have been investigated as a factor influencing measurement results when using real-time PCR technology. This study evaluated the impact of the amplification target, the fluorophore, primer and probe chemistry, and duplexing on measurements by digital PCR to assess their influence on the associated bias.

## 2 Materials and methods

### 2.1 Materials

#### 2.1.1 *In vitro* transcribed (IVT) RNA

*In vitro* transcribed RNA was synthesised in-house, using the pGEM^®^-T easy plasmid vector containing an insert, covering the entire segment 7 (M gene) of influenza A. The insert originated from RNA extracted from influenza A virus A/Aichi/2/68 (H3N2) purchased from the ATCC^®^ Collection (LGC Standards, Molsheim Cedex, France). SuperScript^®^ VILO™ Master Mix (Invitrogen, Carlsbad, CA, USA) was used for cDNA synthesis. The insert was generated using High Fidelity Platinum^®^ PCR SuperMix (Invitrogen) and previously published primers Bm-M-1 and Bm-M-1027R [11]. A standard cloning procedure using pGEM^®^-T Easy Vector System II (Promega Benelux b.v., Leiden, The Netherlands) was carried out according to the manufacturer’s recommendations. Plasmid DNA was extracted from a 5 ml Luria-Bertani (LB) broth culture (grown overnight in a shaking incubator at 37 °C) using The Wizard^®^ Plus SV Minipreps System (Promega) and following the manufacturer’s protocol. The identity of the insert was confirmed by sequencing of both DNA strands (Eurofins MWG Operon, Ebersberg, Germany). *In vitro* transcription was carried out with the RiboMAX™ Large Scale RNA Production System and T7 polymerase (Promega) using *Sal*I (Promega) − linearised plasmid as a template. The transcript was purified using SV Total RNA Isolation System (Promega) according to the producer's protocol, which included treatment with DNase I to remove unwanted DNA from the RNA preparation. The size, purity and integrity of the IVT RNA were confirmed by analysis on the Agilent 2000 Bioanalyzer using the RNA 6000 Pico kit (Agilent Technologies, Diegem, Belgium). Purified IVT RNA was diluted in the RNA Storage Solution (Ambion^®^, Austin, Texas, USA) and 50 μl aliquots were stored at −70 °C. The concentration of IVT RNA was assessed by spectrophotometry (NanoDrop^®^ ND-1000, Wilmington, DE, USA) to be 1.83 × 10^10^ copies/μl. Based on the sequencing results and the position of the T7 promoter, the size of the expected RNA molecule was assessed to be 1147 bp.

#### 2.1.2 Extracted total genomic RNA

Genomic RNA was prepared from cell cultured influenza A virus strain A/Aichi/2/68 (H3N2) propagated in St Georges Hospital, London, and extracted using QIAamp^®^ Viral RNA Mini kit (Qiagen, Inc., Germantown, MD, USA), according to manufacturer's protocol, with minor modifications concerning the elution of the RNA. Samples were eluted in two steps, each using 120 μl RNA Storage Solution (Ambion^®^). Extracted RNA samples were pooled and gently mixed to obtain a homogeneous solution. Twenty and fifty μl aliquots were stored at −70 °C. The concentration of the total genomic influenza RNA was determined by dPCR using 12.765 digital arrays on the BioMark™ HD System (Fluidigm Corporation, San Francisco, CA, USA) according to the published CDC protocol targeting a conserved sequence of the influenza A matrix gene [12]. Three independent aliquots were subjected to dPCR, each measured in triplicate, resulting in an average concentration of 1.27 × 10^6^ copies/μl and relative standard deviation (RSD) of 2.2%.

### 2.2 Methods

#### 2.2.1 Primer and probe chemistry

In total, seven different primer and probe chemistries were applied in this study and are indicated by a specific code in Table 1. ‘DLP-FAM' and ‘DLP-HEX' are both assays using dual labelled probes (DLP) and primers, synthesised according to a published validated qualitative RT-qPCR method by CDC [12]. In DLP-HEX, the fluorescent dye FAM is replaced by a HEX™ fluorophore. Also the primers and probes from 2 other methods, namely ‘DLP-GRAM' and ‘DLP-HA gene', are based on validated methods described by the Pasteur Institute [13] and Yang et al. [14], respectively. The HA gene primers and probe target the H3-haemagglutinin (HA) gene instead of the matrix (M) gene. The primer and probe sequences of the fifth method are identical to the ones of the DLP-FAM and DLP-HEX approaches, but they have been conjugated to repeating spermine derivative cationic units to generate ZNA primers and probe. Scorpions^®^ and LNA primers and probes were designed using the online tool OligoArchitect™ (Sigma-Aldrich, St. Louis, MO, USA). All primers and probes were synthesised and HPLC-purified by Sigma UK, except for ZNAs which were synthesised by Sigma USA. Single-use aliquots of oligonucleotides, reconstituted in nuclease-free water (Promega) were stored at −20 °C. Primer and probe information is included in Table 1 and their position on the M gene can be seen in Fig. S1.

#### 2.2.2 RT-dPCR

dPCR experiments were performed using the 37K IFC Digital Arrays of the BioMark platform (Fluidigm) and the RNA UltraSense™ One-Step Quantitative RT-PCR System (Invitrogen). Quantification of the RNA was done under intermediate precision conditions (independent runs performed on different days) with samples diluted gravimetrically in RNA storage solution buffer (Ambion^®^) and run in triplicate (extracted gRNA) or quintuplicate (IVT RNA). The position of different experiments on the digital array was randomised over three different days. The same sample dilution was used for all the methods on individual days to allow for direct comparison. The digital array was primed and loaded according to manufacturer's protocol. Thermal cycling conditions were: 50 °C for 30 min for reverse transcription, 95 °C for 2 min for denaturation and inactivation of RTase, followed by 45 PCR cycles at 95 °C for 15 s and 55 °C for 30 s [12]. RNA storage solution buffer (Ambion^®^), constituting the no-template control, was included in each experiment. Also a negative control, sonicated human gDNA (Cambio) at 25 ng/μl was analysed to check for unspecific reactions. The analysis was performed utilising the Fluidigm dPCR software version 4.1.2 to assess the concentration by counting the number of positive partitions (H) out of the total number of partitions (C) from which the Poisson distribution was used to estimate the average number of DNA copies per partition (λ) via λ = ln(1-H/C) [15].

Six methods were applied for the analysis of influenza A IVT RNA, consisting of the established RT-PCR methods from CDC [12] and the Pasteur Institute [13], both using dual labelled primers and probe (DLP-FAM and DLP-GRAM), a method with 6-FAM™ replaced by HEX™ fluorophore (DLP-HEX), an experiment with ZNA primers (ZNA), a LNA primers and probe-based method (LNA) and finally a method using Scorpions chemistry (Scorpions^®^). When extracted genomic RNA (gRNA) of influenza A was used as a template, nine different methods were evaluated. In addition to the six methods mentioned above, which are all amplifying particular fragments of the M gene, the extracted gRNA was also quantified with an H3- primer and probe set targeting another gene of the influenza A virus, the haemagglutinin (HA) encoding gene (DLP-HA gene) [14]. Further, the effect of duplexing was evaluated by combining primers and probes targeting the M gene (DLP – HEX) and the HA gene (DLP – HA gene) in a single reaction by using different reporter dyes (FAM *vs.* HEX) [12], [14]. RNA copies in these reaction mixtures were detected by applying two detectors, FAM – MGB and VIC – MGB, with distinct fluorescence spectra.

#### 2.2.3 Optimisation of RT-dPCR: annealing temperature and primer and probe concentrations

Conventional real-time PCR was carried out to determine the optimal annealing temperatures for different primer and probe sets compared in this study. Extracted genomic RNA was diluted volumetrically and used as a template together with the RNA UltraSense™ One-Step Quantitative RT-PCR System (Invitrogen).

The cycling conditions were: 50 °C for 30 min, 95 °C for 2 min, followed by 45 cycles at 95 °C for 15 s and varying annealing temperatures for 30 s [12]. Each assay was run at the following annealing temperatures: 50.0 °C, 51.0 °C, 53.0 °C, 55.9 °C, 59.3 °C, 62.1 °C, 64.1 °C and 65.0 °C. Measurements were performed on the C1000 Touch™ Thermal Cycler (BioRad). RT-PCR products were loaded on a 2 (m/v) % agarose gel (Seakem^®^ LE Agarose, Cambrex Bio Science Rockland, Inc., Rockland, ME, USA) and visualised by gel staining with GelRed™ Nucleic Acid Gel Stain (Biotium, Hayward, CA, USA) and electrophoresis.

To evaluate the influence of different primer and probe concentrations on measurements with the 37K array of the BioMark dPCR system, 8 μl reaction mixtures were used, comprising 3 μl RNA, 1.6 μl RNA UltraSense™ One-Step Quantitative RT-PCR master mix, 0.4 μl 20 x GE sample loading reagent, 0.4 μl RT/Taq mix, 0.16 μl ROX dye and either 0.25 μl, 0.4 μl or 0.6 μl of primers and probes according to the respective primer and probe concentrations investigated. The volume was brought to 8 μl by the addition of nuclease-free water (Ambion^®^). The extracted genomic RNA was diluted 1: 500 to fall within the optimal working interval for dPCR quantification, which is 200–700 positive chambers per panel, corresponding to 230–1900 copies/panel or a λ- value between 0.3 and 2.5 after Poisson correction [16]. Three combinations of primer: probe concentrations, namely (i) 0.8 μmol/l: 0.2 μmol/l (ii) 0.8 μmol/l: 0.3 μmol/l and (iii) 0.5 μmol/l: 0.3 μmol/l were evaluated for 7 methods targeting the M gene [12], [13] or the HA gene [14]. For each method, 3 combinations of primer and probe concentrations were analysed in duplicate and data analysis was done with the Fluidigm Digital PCR Analysis Software version 4.1.2, with manual determination of the fluorescence threshold, the quality threshold (0.05) and the accepted quantification cycle interval (5–45 Cq).

#### 2.2.4 Statistical analysis

The statistical analysis of the measurement data was performed by calculation of descriptive parameters such as mean value, standard deviation (SD) and relative standard deviation (RSD), and by single-factor analysis of variance (ANOVA) in Microsoft Excel 2010. The ANOVA is testing the null hypothesis which assumes that all of the group means in that test are the same. When a significant result from the ANOVA test (*p* value smaller than α) causes the null hypothesis to be rejected, further testing has been performed to determine which pairs of means are significantly different. This was done by Tukey's Honest Significant Differences (HSD), a *Post-hoc* test, where group means are compared pairwise to determine whether the difference between the pair of means is significant. The two means are significantly different if the statistic *q-*value is larger than the critical *q*-value (α, r, dfw), which is based on the probability of error, α (0.05), the number of groups, r, and the degrees of freedom (df_w_) of *MS*_within_, calculated from ANOVA. This value can be obtained from a table of the Studentized range q distribution. The statistic *q-* value is calculated according to Eq. (1):

(1) $$\text{q}=\frac{\left|{\bar{x}}_{1}-{\bar{x}}_{2}\right|}{\sqrt{MS_{\text{within}}/n}}$$

${\bar{x}}_{1}$ mean value of group 1${\bar{x}}_{2}$ mean value of group 2*MS*_within_ mean square within a run from an ANOVA*n* the number of independent replicates per day

For each method, the repeatability (within-run standard deviation, *s*_rep_) and the intermediate precision (between-run standard deviation, *s*_ip_) were derived according to Eq. (2) and Eq. (3):

(2) $$s_{rep}=\sqrt{MS_{\text{within}}}$$

(3) $$s_{ip}=\sqrt{\frac{MS_{\text{between}}\;\;-\;\;MS_{\text{within}}}{n}}$$

*MS*_between_ mean square between runs from an ANOVA.

To assess the variation between the measurements which is solely related to the method, the combined standard uncertainty has been calculated from the uncertainty associated with repeatability, *u*_rep_, and with intermediate precision *u*_ip,_ but no bias related uncertainty was included:

(4) $$\mu =\sqrt{u_{rep}^{2}+u_{ip}^{2}}$$

(5) $$u_{rep}=\sqrt{\frac{MS_{within}}{n}}$$

(6) $$u_{ip}=\sqrt{\frac{\frac{MS_{between}\;\;-\;\;MS_{within}}{n}}{N}}$$

*N* the number of measurement days.

To obtain a relative standard uncertainty, the combined standard uncertainty, *u*_,_ is divided by the mean value of the measurements.

## 3 Results and discussion

### 3.1 Optimisation of RT-dPCR

To optimise the RT-dPCR reaction for the different methods compared, a temperature gradient experiment was performed with conventional real-time PCR, using a hundredfold diluted extracted gRNA and the RNA UltraSense™ One-Step Quantitative RT-PCR System (Invitrogen). As specified in the methods chapter above, samples were subjected to 8 different annealing temperatures in the range of 50 °C–65 °C and final reaction products were visualized by gel electrophoresis. Fig. S2 shows the gel images of this experiment for the primer and probe sets (number 1–6) tested. Visual inspection of the gel images showed single amplification products for all sets, run at different annealing temperatures, except for locked nucleic acid (LNA) primers and probe. Although an optimal annealing temperature of 65 °C for LNA probes has been previously reported to be useful for more sensitive DNA detection [10], no amplification product could be observed above 62 °C in our experiment. Based on these findings and in line with the published conditions for the CDC measurement procedure [12], an annealing temperature of 55 °C was chosen for all the RT-dPCR experiments.

A second optimisation experiment was performed using dPCR to determine the optimal concentration of the different primer and probe sets used. For none of the methods, significant differences between the various primer and probe concentrations could be observed, except for the ZNA primer and probe set. The highest copy numbers per microliter were obtained for the ZNA primer: probe concentration of 0.5 μmol/l: 0.3 μmol/l, while only 14–28% of the RNA copies could be recovered when a higher primer concentration was used (*p* < 0.001) (Fig. S3). According to the data obtained, a final concentration of primers and probes of 0.8 μmol/l and 0.2 μmol/l, respectively, was selected for all methods, except for the ZNA method, which will be used at a primer concentration of 0.5 μmol/l and probe concentration of 0.3 μmol/l.

### 3.2 Factors influencing RNA quantification with RT-dPCR

#### 3.2.1 Influence of primer and probe chemistry

**Fig. 1:**
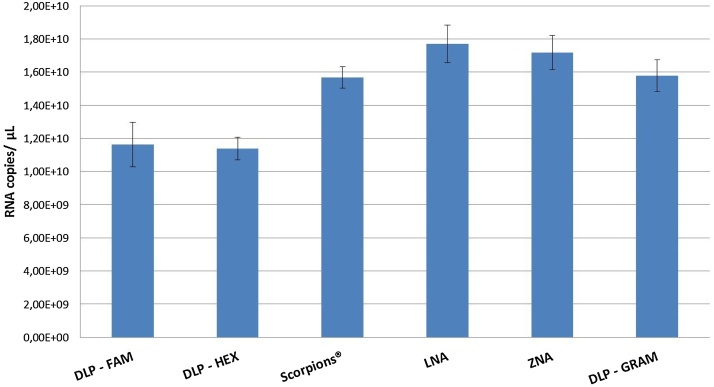
RT-dPCR quantification of IVT viral RNA using different primer and probe chemistries. Each column represents average RNA copy number/μl obtained in 3 independent experiments with 5 replicate reactions. Error bars indicate the standard uncertainty of the intermediate precision calculated according to Eq. (3) using ANOVA (between- and within-run variance).

**FIG. 2:**
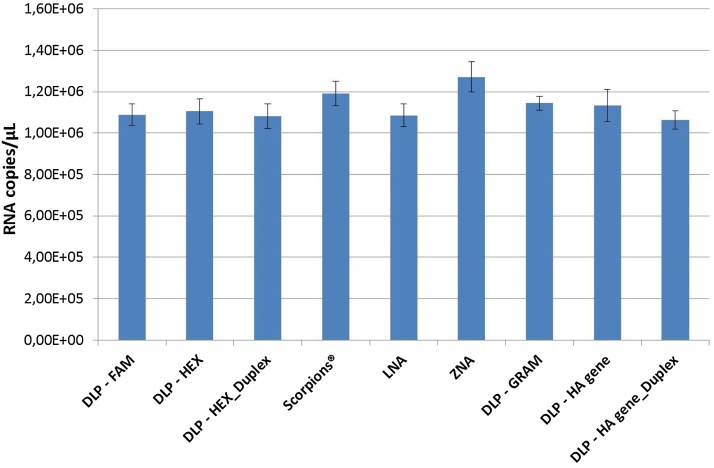
RT-dPCR quantification of extracted genomic viral RNA using different primer and probe chemistries. Each column represents average RNA copy number/μl obtained in 3 independent experiments with 3 replicate reactions. Error bars indicate the standard uncertainty of the intermediate precision calculated according to Eq. (3) using ANOVA (between- and within-run variance).

The influence of different primer and probe chemistries on RT-dPCR quantification was investigated for two RNA template types, a small (1147 bp) *in vitro* transcribed RNA fragment (IVT RNA) and total genomic RNA extracted from Influenza A virus. Fig. 1 shows the average RNA copy numbers obtained in experiments with IVT RNA, while results obtained with extracted RNA are presented in Fig. 2. More detailed results are shown in Tables S1–S3. Six different measurement methods were run with primer and probe concentrations previously determined as optimal, consisting of two methods using conventional dual labelled probes conjugated with FAM fluorophore and BHQ1 non-fluorescent quencher, DLP – FAM [12] and DLP-GRAM [13], one DLP method with HEX™ fluorophore instead of 6-FAM™, DLP – HEX, a LNA approach, a ZNA technology, and a method using Scorpions technology. Fig. 1 shows that the LNA method resulted in the highest average RNA copy number values measured (1.77 × 10^10^ copies/μl), followed by ZNA (1.72 × 10^10^ copies/μl), the conventional DLP − GRAM method (1.58 × 10^10^ copies/μl), and Scorpions^®^ (1.57 × 10^10^ copies/μl) technology. According to results from the HSD Tukey's test, the difference between the average RNA copy numbers obtained with the LNA and ZNA methods, targeting the same region of the Influenza A matrix gene were not statistically significant, neither were the differences between Scorpions^®^ and DLP – GRAM [13], targeting another region of the M gene more upstream (Table S3). However, a significant increase in the measured RNA copy number was observed in this experiment when the ZNA primers and probe were used in comparison with its unmodified counterpart, DLP – FAM [12] and this for both RNA templates (Fig. 1, FIG. 2), demonstrating the beneficial use of more sophisticated primers and probe. However, this study does not allow to distinguish at which step of the RT-PCR these probe types have a positive impact.

The finding that the **LNA method** is associated with higher estimates of concentration when measuring IVT RNA, is in accordance with previous studies, demonstrating superior performance of LNA primer and probes in terms of efficiency and sensitivity [8], [10]. This increased sensitivity is caused by the incorporation of LNA monomers in the primer and probe sequence. The sugar phosphate backbone of a LNA monomer has a 2′-O, 4′-C methylene bridge, hereby introducing a conformational lock of the molecule. This enhances the monomer’s thermal stability, reduces its flexibility, and thus increases the hybridisation performance of LNA containing probes compared to classical dual labelled probes [17]. Furthermore, a shorter primer and probe can be designed. This LNA-associated enhancement of hybridisation is most probably through improved base stacking interactions and hydrogen bonds [18].

Another approach to improve the nucleic acid binding affinity is to decrease the electrostatic repulsion between negatively charged nucleic acid strands. Based on this electrostatic interaction mechanism the **Zip Nucleic Acids (ZNA)** method was developed. ZNA are oligonucleotides conjugated with spermine residues as cationic units to reduce the negative charge and favour hybridisation with a complementary sequence by clipping the strands together like a zipper, hence their name Zip Nucleic Acids [9]. In this study, the largest influenza A RNA copy numbers were obtained with ZNA primers and probe, independent of the template type, which demonstrate the high target affinity of such oligonucleotides and their ability to improve the efficiency of the reversed transcription reaction of RT-PCR, leading to increased sensitivity [9].

A third alternative probe chemistry which has been investigated in this study is **Scorpions^®^,** a method wherein primer and probe are combined into a single molecule. A Scorpion consists of a specific probe sequence held in a hairpin loop conformation by complementary stem sequences on both ends of the probe. The probe contains a 5′-end reporter dye and a 3′-end quencher dye directly linked to the 5′-end of a PCR primer via a PCR blocker. The PCR blocker prevents that the PCR primer is further extended, which could lead to the opening of the hairpin loop in the absence of the specific target and to the detection of non-specific PCR products [19]. The bi-molecular Scorpion consists of a fluorescently labelled probe coupled to a primer by a PCR blocker and linked to a second oligonucleotide that is complementary to the probe sequence and has a quencher at the 3′-end. The mechanism of action is similar to that of the unimolecular probe [19], [20]. The unimolecular mechanism which characterises the Scorpions^®^ chemistry is kinetically favourable and highly efficient due to the direct binding of the probe to the target sequence, ensuring that each probe has a target in the near vicinity. Enzymatic cleavage is not required, which improves the PCR reaction time significantly in comparison with conventional dual labelled probes. There is a direct relationship between the number of amplicons generated and the emitted fluorescence signal [19]. Although a publication from Thellwell et al. [20] reported a better performance of Scorpions^®^ primers when compared to dual labelled probes due to its unimolecular probing mechanism, our data did not confirm this expected higher sensitivity compared to the conventional DLP method targeting the same region of the M gene, DLP-GRAM (Fig. 1 and Table S3).

However, our findings are in line with those of another comparative study performed by Reynisson et al. [10]. It should be noted that the design of the Scorpions^®^ probe tested in the various studies was not always the same. In the study from Thellwell et al. [20], the probe was designed from the Scorpions^®^ primer by removing the stem sequences and adding the quencher and reporter to the resulting probe sequence. In this study and the one of Reynisson et al. [10] the Scorpions^®^ primer and dual label probes were both designed as independent, new probe sequences. Also noteworthy is that the Scorpions^®^ method, together with ZNA, generated the strongest PCR response when extracted RNA was used as a template (Fig. 2, Table S1-B).

Significant differences in RNA yield could be observed when IVT RNA was quantified with more sophisticated primer and probe chemistries compared to the conventional dual labelled probes. It should be noted that these differences are less than twofold and such differences may be tolerable in daily routine for applications such as diagnostics of infectious diseases which widely apply presence-absence testing. However, for standardisation purposes these differences are important when the trueness and the uncertainty of a measurement result (including uncertainties associated with the assessment of bias) needs to be assessed. When RT-dPCR is applied to assign values to reference materials, a bias should be as low as possible or ideally eliminated. It is known that RT-dPCR introduces a larger variability into the measurement results than dPCR on its own [6]. This is due to the extra PCR step, converting RNA into cDNA, which could be subject to variable transcription efficiency, e.g. depending on the selected reverse transcriptase. The additional step also enlarges the chance of a so-called amplification drop out. This phenomenon is defined as the failure of amplification of a target molecule present in the dPCR partition [6]. Several factors could be responsible for an amplification drop out, including inhibition compounds in the sample (matrix effect), template molecular complexity, reagent inhomogeneity and also primer and probe chemistry. Different extraction methods and (one step) RT-PCR master mixes have been evaluated in a separate study which demonstrated that they can have a major effect on the bias (data not shown). The optimal extraction method (in terms of extraction efficiency and matrix effects) and RT-PCR master mix (in terms of minimal amplification drop out) have been applied for the purpose of this study. Both, variable transcription efficiency and amplification drop out are important sources of negative bias.

#### 3.2.2 Influence of dPCR amplification target

Besides the influence of the dPCR template type and the primer and probe chemistry, the choice of the target may also have an effect on dPCR measurements. When comparing the results obtained with the classical dual labelled primer and probes, targeting different regions of the same influenza A matrix gene, DLP- FAM and DLP – HEX versus DLP-GRAM, or even of a different gene (DLP-HA gene), no significant difference could be found for the extracted gRNA template (Fig. 2). However, when IVT RNA was quantified, significantly lower RNA copy numbers have been observed for DLP-FAM and DLP-HEX, regardless of the fluorophore used (p < 0.000001) (Fig. 1 and Table S3A). The replacement of the fluorophore from 6-FAM™ to HEX™ had no significant effect on the quantitative results (IVT RNA: 1.16 × 10^10^ and 1.14 × 10^10^ copies/μl, respectively and extracted gRNA: 1.09 × 10^6^ and 1.10 × 10^6^ copies/μl, respectively).

#### 3.2.3 Influence of dPCR template type

Larger variations were observed between RNA concentrations obtained with different primer and probe chemistries when an IVT RNA fragment was targeted (RSD of 18%) compared to the extracted genomic RNA template (RSD of 9%). The results from the HSD Tukey's test revealed important differences between the two RNA template types. For IVT RNA, twelve out of fifteen (resembling 80%) method combinations were significantly different, while only six out of thirty-six (resembling 17%) of the combinations for extracted genomic RNA were found to provide significantly different RNA copy number concentrations (Table S3). The significant increase in RNA copy numbers observed when the ZNA primers and probe were used in comparison with its unmodified counterpart, DLP – FAM, was more than 32% for IVT RNA, while this large difference was mitigated to 14% when extracted gRNA was used as a template. Further, a 36% difference was measured between the highest and lowest measurement results for IVT RNA, while the difference between the maximum and minimum values obtained for extracted gRNA was only 16%.

This interesting different behaviour of two nucleic acid template types has also been described for quantitative dPCR data of DNA, for which the more complex secondary structure of the genomic DNA and the associated higher molecular dropout was seen as an important factor for the greater variance observed with purified genomic DNA as template [16], [21]. However, in this study, larger variation was seen for a single RNA fragment obtained by *in vitro* synthesis and not for the longer, more complex genomic RNA isolated from influenza A virus cell culture. A possible explanation could be that the smaller IVT RNA is less stable than the gRNA template. Higher affinity and, consequently, more efficient hybridisation of the modified oligonucleotides, such as ZNA or Scorpions, could result in faster reaction kinetics allowing for generation of cDNA before any degradation of the RNA sample occurs. Further studies are needed to confirm this hypothesis.

#### 3.2.4 Influence of duplexing

It should also be noted that there was almost no effect of duplexing. During duplexing the measurement methods targeting the M gene and HA gene were combined in a single reaction and compared to the detection of the respective genes in separate methods (Fig. 2, Table S3-B).

These data show that the impact of different primer and probe chemistries depends on the template used and emphasise the importance of method design and optimisation, before considering any additional modifications of primers and probes. Depending on the template type and performance requirements of the analysis, potential advantages of more sophisticated approaches may not substantiate additional costs. In case of assigning values to reference materials, some of these modified synthetic oligonucleotides could be an interesting option, although this should be evaluated for the target and template of interest on a case-by-case basis.

#### 3.2.5 Precision

Having compared the different primer and probe chemistries and their influence on RT-dPCR measurements, the precision of the dPCR analyses was evaluated under conditions of intermediate precision where replicate measurements were performed on separate days within the same laboratory [22].

When estimating the combined standard uncertainty for the precision of a measurement procedure, it is necessary to assess the repeatability (within-run), *s*_rep_ and the intermediate precision (between-run), *s*_ip_. Five replicate measurements were performed per method for IVT RNA and this in three independent runs, each on a different day. For gRNA extracted from virus culture, measurements were done in triplicate on each of 3 different days. Uncertainties were calculated based on *s*_rep_ and *s*_ip_ according to the equations as given above. No bias related uncertainty has been included.

When measurements were performed on IVT RNA, relative combined standard uncertainties were on average 3.4%, with all of the methods having uncertainties <5% except for the method using a conventional dual labelled probe and fluorophore FAM (DLP-FAM), for which 6.3% was estimated. The majority of this larger standard uncertainty could be attributed to an increased value of 10% for the intermediate precision (Table 2(A)).

The methods with the strongest PCR response for IVT RNA (ZNA and LNA) had a comparable combined standard uncertainty of about 3%. These low uncertainty values could be confirmed when extracted gRNA was used as a template, resulting in uncertainties from 2.9% (DLP – GRAM) to 6.8% for the DLP – HA gene method. This slightly higher uncertainty value of the DLP – HA gene method was due to an intermediate precision of 11.4%, whereas the intermediate precision for the DLP – GRAM method was much better with 1.4%. The average relative standard uncertainty for measurements on the extracted genomic RNA template was found to be 5%. Overall, the standard uncertainty of the values measured, independent of the method or sample template evaluated, was ≤6.8%, which illustrates the ability of RT-dPCR to quantify viral RNA in a precise and reproducible manner, without the need for calibration with a matched nucleic acid template.

## 4 Conclusions

Although the theory of dPCR assumes that each target nucleic acid molecule present in a partition of the BioMark dPCR chip (Fluidigm) will undergo successful amplification, this study and many others have shown that more careful method design and optimisation are required to obtain an accurate quantitative result [6], [7], [23], [24]. The uncertainties observed demonstrate that precise and reproducible quantification of extracted genomic- as well as synthetic RNA is achievable by RT-dPCR. However, this high precision also led to the observation of significant bias between RNA measurements when different methods were applied. Pronounced advantages could be found when more sophisticated primer and probe chemistries, such as ZNA and LNA chemistries, were used. Also, the type of RNA template had a significant influence on the outcome of the RNA quantification.

This study has also shown that the replacement of the fluorophore from FAM- to HEX- did not have any impact on the obtained RNA copy numbers. Further, no effect on the RNA concentration has been found for the simultaneous detection of two different genes in duplex test format, compared to the result of their single-plex partners. However, targeting different amplification regions may result in differences in RNA or DNA concentration, which could be of major importance, depending on the application of the measurement results.

In studies where RNA reference materials intended for RT-qPCR calibration would be characterised by RT-dPCR, approaches as applied in this study should be taken into account to ensure value assignment with minimal negative bias due to amplification drop out. It is important to emphasise that, based on the limited studies carried out so far, and in line with the statement of Josefsen et al. [8], general conclusions cannot be drawn from a single study. The fact that two different template types, both of which are typical calibration materials, were not equally affected by applying different assay chemistries could potentially lead to systematic errors when RNA is quantified in more complex clinical samples.

Overall, this study demonstrates that RT-dPCR, when sufficiently well optimised, has enormous potential for the accurate quantification of viral RNA. However, significant bias can be observed when various target sequences and primer and probe chemistries are applied, reinforcing the importance of careful evaluation of the RT- dPCR method performance and quality control measures.

## Figures and Tables

**Table 1:** Primers and probes for detection of influenza A viral RNA by RT- dPCR.

Method	Primer/probe	Target	Sequence (5′-3′)	Tm (°C)	Label	Amplicon (bp)	Refs.
DLP – FAM	InfA Forward^a^	Matrix gene (M)	GACCAATCCTGTCACCTCTGAC	64.9	NA	106	Modified from CDC [12]
	InfA Reverse^a^		AGGGCATTTTGGACAAAGCGTCTA	70.1	NA		
	InfA Probe		TGCAGTCCTCGCTCACTGGGCACG	80	FAM/BHQ1		

DLP – HEX	InfA Forward^a^	Matrix gene (M)	GACCAATCCTGTCACCTCTGAC	64.9	NA	106	Modified from CDC [12]
	InfA Reverse^a^		AGGGCATTTTGGACAAAGCGTCTA	70.1	NA		
	InfA_probe		TGCAGTCCTCGCTCACTGGGCACG	80	HEX/BHQ1		

Scorpions^®^	ScFor	Matrix gene (M)	GCCTTCTAACCGAGGTCGAAACG	70.5		79	This study
	ScProbe		GGTCACCGTCTCTCTATCGTCCCGTCAGGCCGGTGACC-BHQ1-HEG-AGTCTCTGTGCGATCTCGGCTTT	93.8	FAM/BHQ1		

LNA	LNA_For	Matrix gene (M)	CTCTCATGGAATGGCTAA	56.1	NA	74	This study
	LNA_Rev		CGTGAATACAAATCCCAAA	58.4	NA		
	LNA_Probe		cca(+A)tc(+C)tg(+T)ca(+C)ctct	59.9	FAM/BHQ1		

ZNA	ZNA_InfA_F	Matrix gene (M)	(Zbase)(Zbase)(Zbase)(Zbase)GACCAATCCTGTCACCTCTGAC	64.9	NA	106	Modified from CDC [12]
	ZNA_InfA_R		(Zbase)(Zbase)(Zbase)(Zbase)AGGGCATTTTGGACAAAGCGTCTA	70.1	NA		
	ZNA_InfA_P		(Zbase)(Zbase)(Zbase)(Zbase)TGCAGTCCTCGCTCACTGGGCACG	80	FAM/BHQ1		

DLP – GRAM	GRAM/7Fw	Matrix gene (M)	CTTCTAACCGAGGTCGAAACGTA	65.1	NA	202	Pasteur Institute protocol
	GRAM/161Rv		GGTGACAGGATTGGTCTTGTCTTTA	66.1	NA		
	GRAM probe/52/+		TCAGGCCCCCTCAAAGCCGAG	75.4	FAM/BHQ1		

DLP – HA gene	H3-F^a^	H3-haemagglutinin gene (HA)	ACCAGAGAAACAAACTAGAGGCCTATT	65	NA	120	Yang et al. [14]
	H3-R		TGTCCTGTGCCCTCAGAATTT	65.8	NA		
	H3-P		CGGTTGGTACGGTTTCAGGCA	71	FAM/BHQ1		

NA – not applicable; FAM – 6-carboxyfluorescein; HEX – hexachlorofluorescein; BHQ1 – Black Hole Quencher^®^-1 dye; ZNA – zip nucleic acid; LNA – locked nucleic acid; HEG – hexethylene glycol reverse – extension blocker.

a Primers modified according to the sequence of the influenza strain used.

**Table 2:** Calculation of the relative standard uncertainties based on the repeatability and intermediate precision of RNA measurements by RT-dPCR for A) IVT RNA template and B) extracted genomic RNA template.

(A) *IVT RNA*	DLP – FAM	DLP – HEX	Scorpions^®^	LNA	ZNA	DLP – GRAM
Repeatability (within-run standard deviation s_rep_)	**5.9%**	**5.4%**	**2.8%**	**5.8%**	**5.0%**	**4.9%**
Intermediate precision (between-run standard deviation *s*_ip)_	**10.0%**	**2.3%**	**3.0%**	**2.8%**	**3.3%**	**3.6%**
Uncertainty related to *s*_rep_	**2.7%**	**2.4%**	**1.2%**	**2.6%**	**2.2%**	**2.2%**
Uncertainty related to *s*_ip_	**5.7%**	**1.3%**	**1.7%**	**1.6%**	**1.9%**	**2.1%**
**Combined uncertainty *u***	****6.3%****	****2.8%****	**2.1%**	**3.1%**	**2.9%**	**3.0%**
**Average combined uncertainty *u***	****3.4%****					

(B) *Extracted gRNA*	DLP – FAM	DLP – HEX	DLP – HA gene_Duplex	DLP – HEX_Duplex	Scorpions^®^	LNA	ZNA	DLP – GRAM	DLP – HA gene
Repeatability (within-run standard deviation *s*_r_)	**4.8%**	**4.1%**	**4.7%**	**3.0%**	**6.8%**	**4.7%**	**5.6%**	**4.8%**	**2.9%**
Intermediate precision (between-run standard deviation *s*_ip)_	**6.5%**	**8.5%**	**5.7%**	**8.9%**	**5.3%**	**7.4%**	**8.1%**	**1.4%**	**11.4%**
Uncertainty related to *s*_r_	**2.8%**	**2.4%**	**2.7%**	**1.7%**	**3.9%**	**2.7%**	**3.2%**	**2.8%**	**1.7%**
Uncertainty related to *s*_ip_	**3.8%**	**4.9%**	**3.3%**	**5.1%**	**3.1%**	**4.3%**	**4.7%**	**0.8%**	**6.6%**
**Combined uncertainty *u***	**4.7%**	**5.5%**	**4.3%**	**5.4%**	**5.0%**	**5.1%**	**5.7%**	**2.9%**	**6.8%**
**Average combined uncertainty *u***	**5.0%**								

## References

[1] Baker M. (2012). Digital PCR hits its stride. Nat. Methods.

[2] Corbisier P., Bhat S., Partis L., Xie V.R., Emslie K.R. (2010). Absolute quantification of genetically modified MON810 maize (Zea mays L.) by digital polymerase chain reaction. Anal. Bioanal. Chem..

[3] Haynes R.J., Kline M.C., Toman B., Scott C., Wallace P., Butler J.M., Holden M.J. (2013). Standard reference material 2366 for measurement of human cytomegalovirus DNA. J. Mol. Diagn..

[4] White H., Deprez L., Corbisier P., Hall V., Lin F., Mazoua S., Trapmann S., Aggerholm A., Andrikovics H., Akiki S., Barbany G., Boeckx N., Bench A., Catherwood M., Cayuela J.M., Chudleigh S., Clench T., Colomer D., Daraio F., Dulucq S., Farrugia J., Fletcher L., Foroni L., Ganderton R., Gerrard G., Gineikienė E., Hayette S., El Housni H., Izzo B., Jansson M., Johnels P., Jurcek T., Kairisto V., Kizilors A., Kim D.W., Lange T., Lion T., Polakova K.M., Martinelli G., McCarron S., Merle P.A., Milner B., Mitterbauer-Hohendanner G., Nagar M., Nickless G., Nomdedéu J., Nymoen D.A., Leibundgut E.O., Ozbek U., Pajič T., Pfeifer H., Preudhomme C., Raudsepp K., Romeo G., Sacha T., Talmaci R., Touloumenidou T., Van der Velden V.H., Waits P., Wang L., Wilkinson E., Wilson G., Wren D., Zadro R., Ziermann J., Zoi K., Müller M.C., Hochhaus A., Schimmel H., Cross N.C., Emons H. (2015). A certified plasmid reference material for the standardisation of BCR-ABL1 mRNA quantification by real-time quantitative PCR. Leukemia.

[5] Hayden R.T., Gu Z., Sam S.S., Sun Y., Tang L., Pounds S., Caliendo A.M. (2015). Comparative evaluation of three commercial quantitative cytomegalovirus standards using digital and real-time PCR. J. Clin. Microbiol..

[6] Sanders R., Mason D.J., Foy C.A., Huggett J.F. (2013). Evaluation of digital PCR for absolute RNA quantification. PLoS One.

[7] Devonshire A.S., Honeyborne I., Gutteridge A., Whale A.S., Nixon G., Wilson P., Jones G., McHugh T.D., Foy C.A., Huggett J.F. (2015). Highly reproducible absolute quantification of Mycobacterium tuberculosis complex by digital PCR. Anal. Chem..

[8] Josefsen M.H., Löfström C., Sommer H.M., Hoorfar J. (2009). Diagnostic PCR: comparative sensitivity of four probe chemistries. Mol. Cell. Probes.

[9] Moreau V., Voirin E., Paris C., Kotera M., Nothisen M., Rémy J.S., Behr J.P., Erbacher P., Lenne-Samuel N. (2009). Zip nucleic acids: new high affinity oligonucleotides as potent primers for PCR and reverse transcription. Nucleic Acids Res..

[10] Reynisson E., Josefsen M.H., Krause M., Hoorfar J. (2006). Evaluation of probe chemistries and platforms to improve the detection limit of real-time PCR. J. Microbiol. Methods.

[11] Hoffmann E., Stech J., Guan Y., Webster R.G., Perez D.R. (2001). Universal primer set for the full-length amplification of all influenza A viruses. Arch. Virol..

[12] World health Organisation, The WHO Collaborating Centre for influenza at CDC Atlanta, United States of America. CDC protocol of realtime RT-PCR for swine influenza A (H1N1). 28 April 2009, revision 1 (30 April 2009), revision 2 (6 October 2009). CDC REF. # I-007-05. http://www.who.int/csr/resources/publications/swineflu/CDCrealtimeRTPCRprotocol_20090428.pdf.

[13] Sylvie van der Werf, Dominique Rousset, Vincent Enouf from Institut Pasteur. National Influenza Center (Northern-France) – Unit of Molecular Genetics of RNA Viruses. Real-time PCR of Type A A(H1N1)2009 viruses. Ref: SOP/FluAswl/031209. http://social-sante.gouv.fr/IMG/pdf/Protocoles_CNR_03122009.pdf.

[14] Yang Y., Gonzalez R., Huang F., Wang W., Li Y., Vernet G., Wang J., Jin Q. (2010). Simultaneous typing and HA/NA subtyping of influenza A and B viruses including the pandemic influenza A/H1N1 2009 by multiplex real-time RT-PCR. J. Virol. Methods.

[15] Dube S., Qin J., Ramakrishnan R. (2008). Mathematical analysis of copy number variation in a DNA sample using digital PCR on a nanofluidic device. PLoS One.

[16] Whale A.S., Cowen S., Foy C.A., Huggett J.F. (2013). Methods for applying accurate digital PCR analysis on low copy DNA samples. PLoS One.

[17] J. Wengel, Locked Nucleic Acid Technology^TM^ : A brief overview. Available from: https://www.exiqon.com/ls/Documents/Scientific/Locked%20Nucleic%20Acid%20Technology%20a%20brief%20overview.pdf.

[18] Arora A., Kaur H., Wengel J., Maiti S. (2008). Effect of locked nucleic acid (LNA) modification on hybridization kinetics of DNA duplex. Nucleic Acids Symp. Ser..

[19] Sigma qPCR Technical Guide. http://www.gene-quantification.de/SIAL-qPCR-Technical-Guide.pdf.

[20] Thelwell N., Millington S., Solinas A., Booth J., Brown T. (2000). Mode of action and application of Scorpion primers to mutation detection. Nucleic Acid Res..

[21] Bhat S., Herrmann J., Armishaw P., Corbisier P., Emslie K.R. (2009). Single molecule detection in nanofluidic digital array enables accurate measurement of DNA copy number. Anal. Bioanal. Chem..

[22] International Organisation for Standardisation, ISO 5725-3:1994, Accuracy (trueness and precision) of measurement methods and results – Part 3: intermediate measures of the precision of a standard measurement method, Geneva.

[23] Sanders R., Huggett J.F., Bushell C.A., Cowen S., Scott D.J., Foy C.A. (2011). Evaluation of digital PCR for absolute DNA quantification. Anal. Chem..

[24] Huggett J.F., Cowen S., Foy C.A. (2015). Considerations for digital PCR as an accurate molecular diagnostic tool. Clin. Chem..

