# Supplementary material for: Influence of primer & probe chemistry and amplification target on reverse transcription digital PCR quantification of viral RNA
Source: Biomol Detect Quantif. 2016 Aug 27;9:20–8. doi: 10.1016/j.bdq.2016.08.003 (PMC5007883; doi:10.1016/j.bdq.2016.08.003)
Supplement: Supplementary file 1 [file mmc1.docx]

**Supplementary Information:**

**SUPPLEMENTAL FIGURES**

**
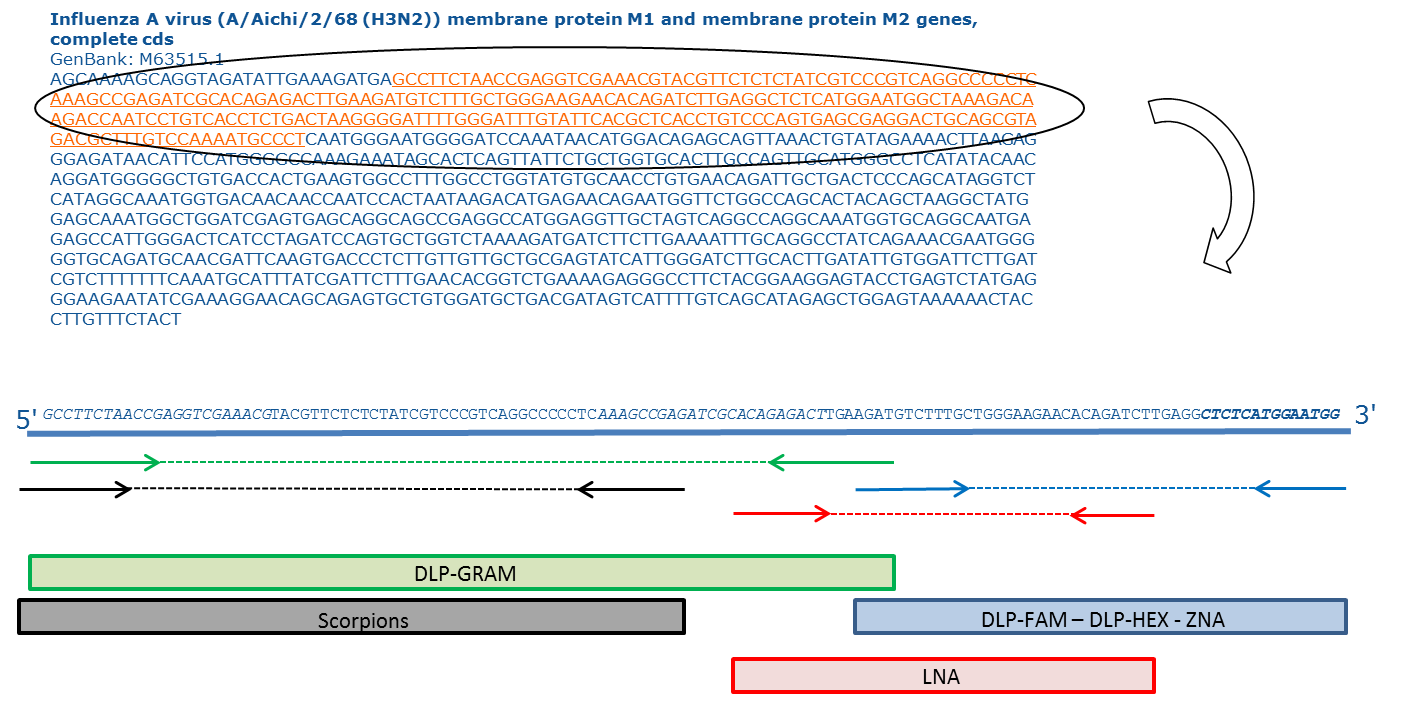
**

**Figure S1:** DNA sequence of the membrane proteins, M1 and M2 of the influenza A virus strain A/Aichi/2/68 (H3N2) (PubMed Genbank accession number M63515.1) and the schematic position of the 6 different primer pairs evaluated for the M gene. Primer pairs and their amplified fragments are indicated by colours, corresponding to the different methods. DLP-GRAM is indicated in green, Scorpions primers and amplicon in black, LNA in red and the fragment amplified by DLP-FAM, DLP-HEX and ZNA chemistries is shown in blue.

| 1. DLP - FAM  A B C D E F G H  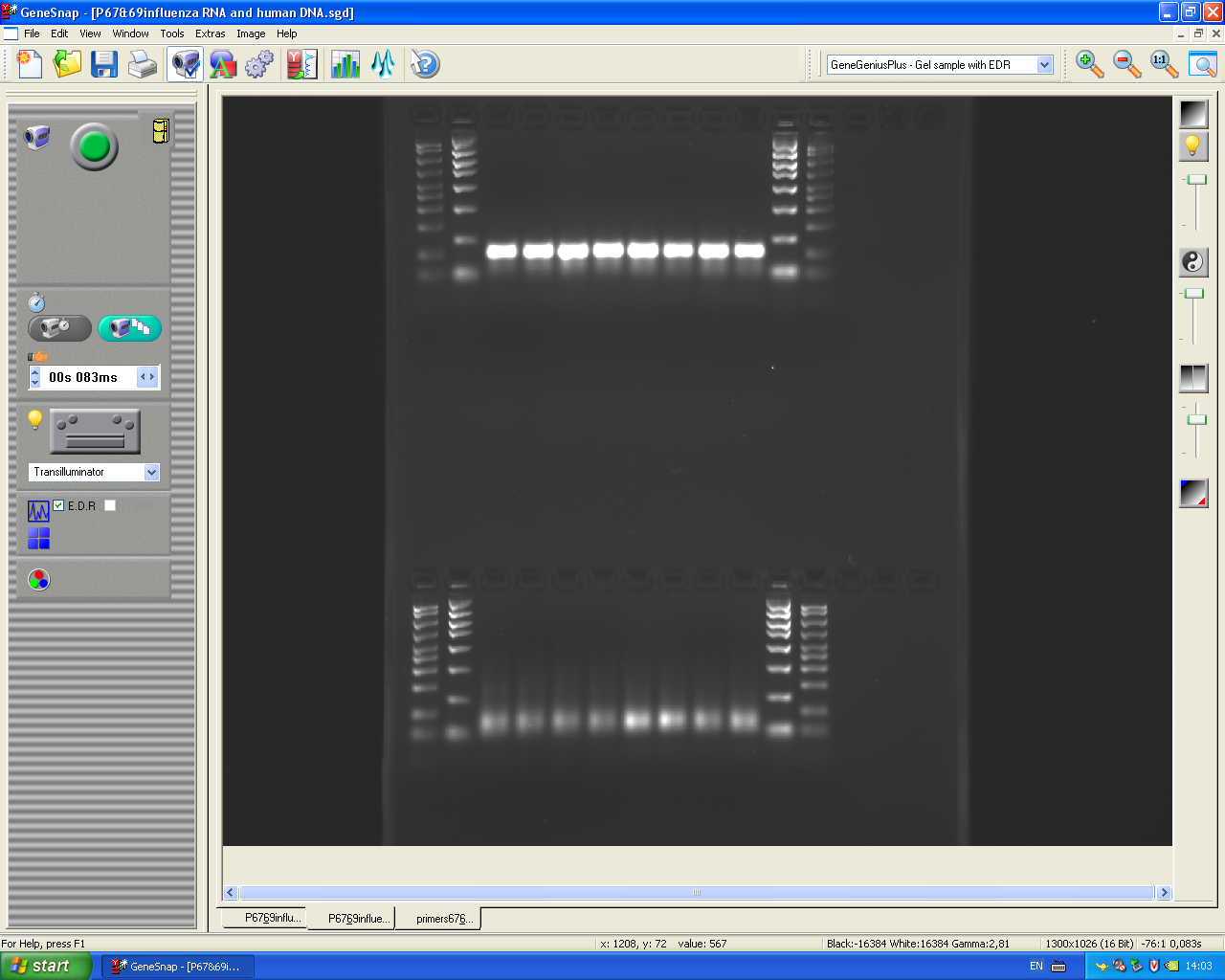 | 2. Scorpions^®^ primers  A B C D E F G H  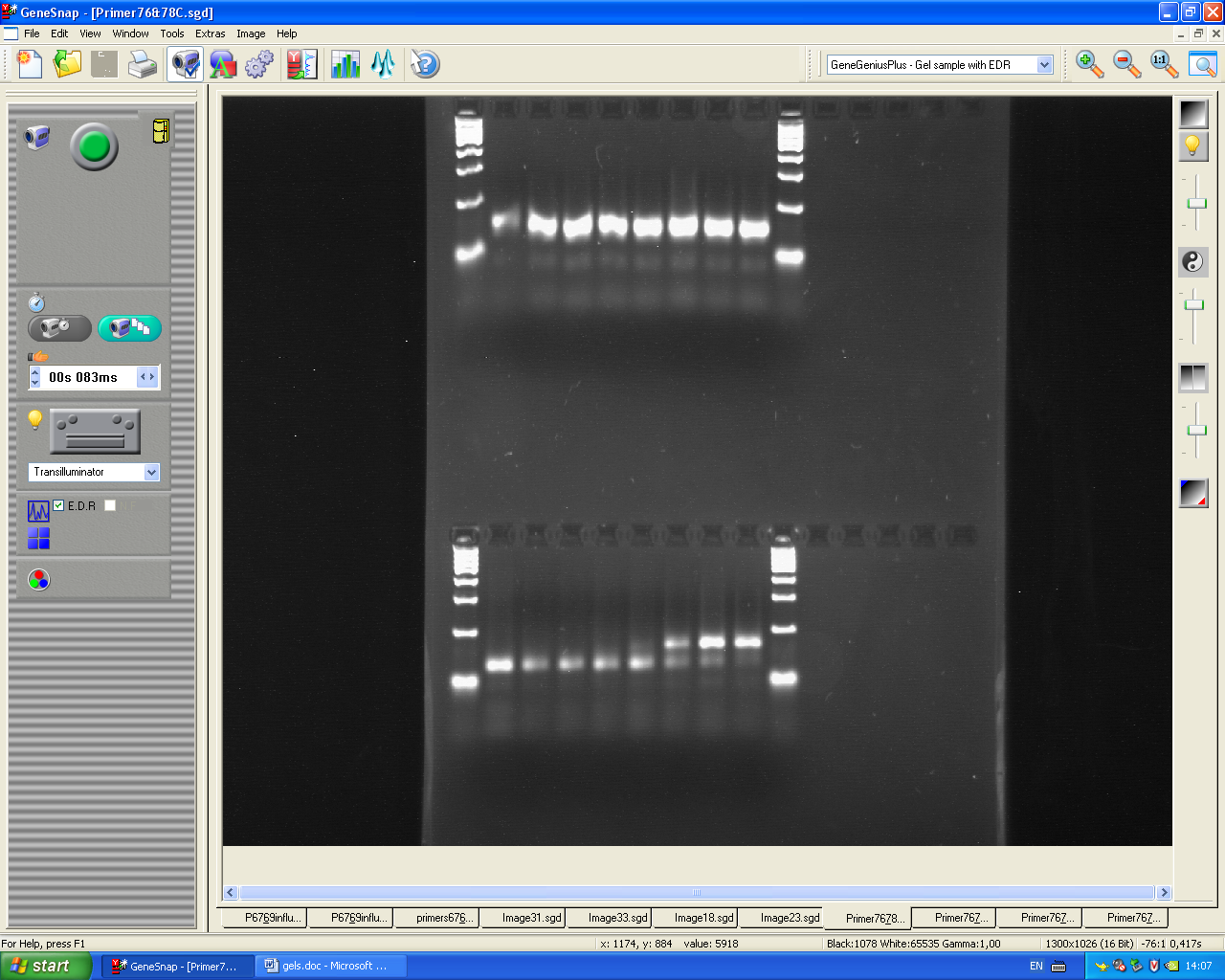 | 3. Locked nucleic acid (LNA) primers  A B C D E F G H  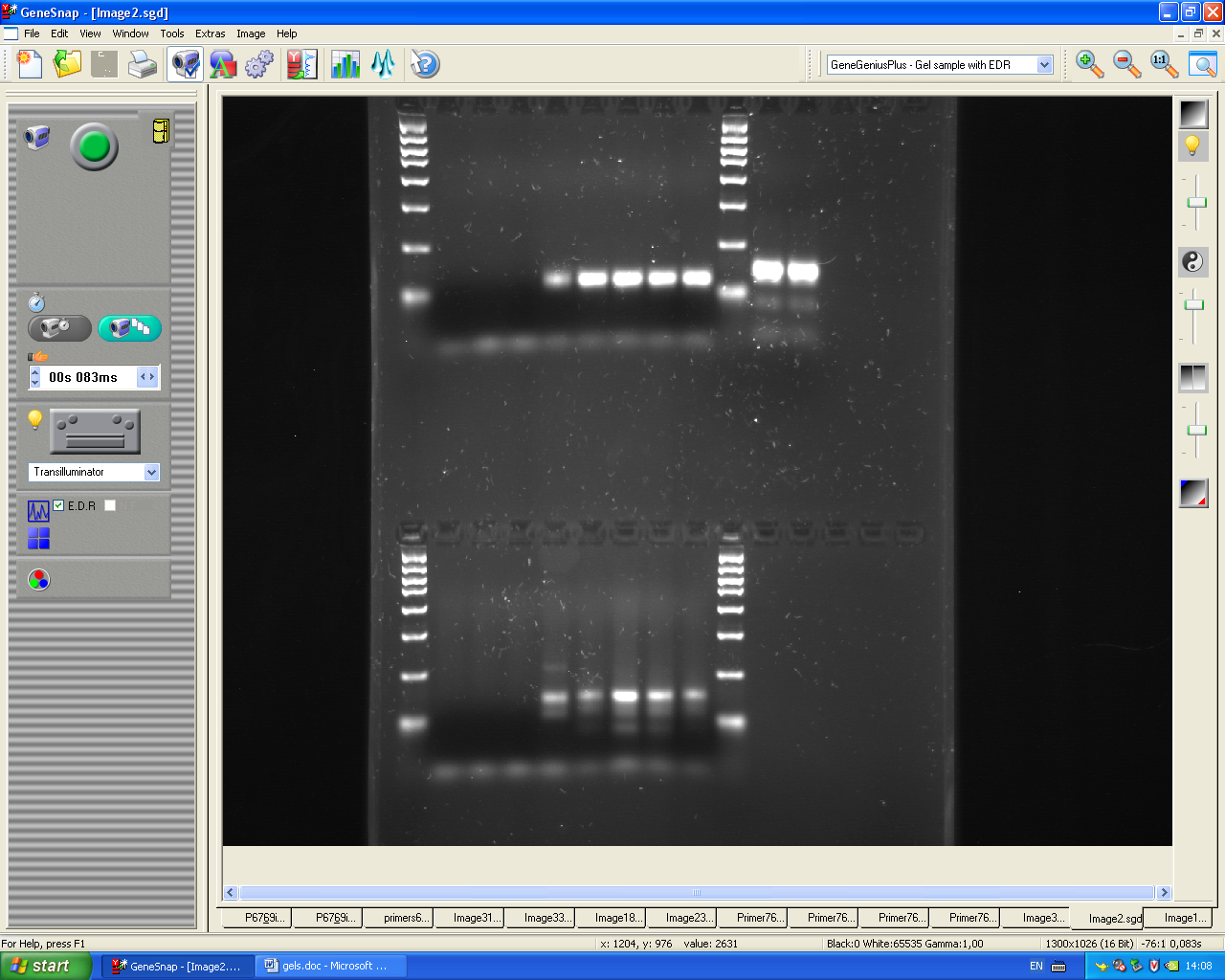 |
| --- | --- | --- |
| 4. DLP – HA gene  A B C D E F G H  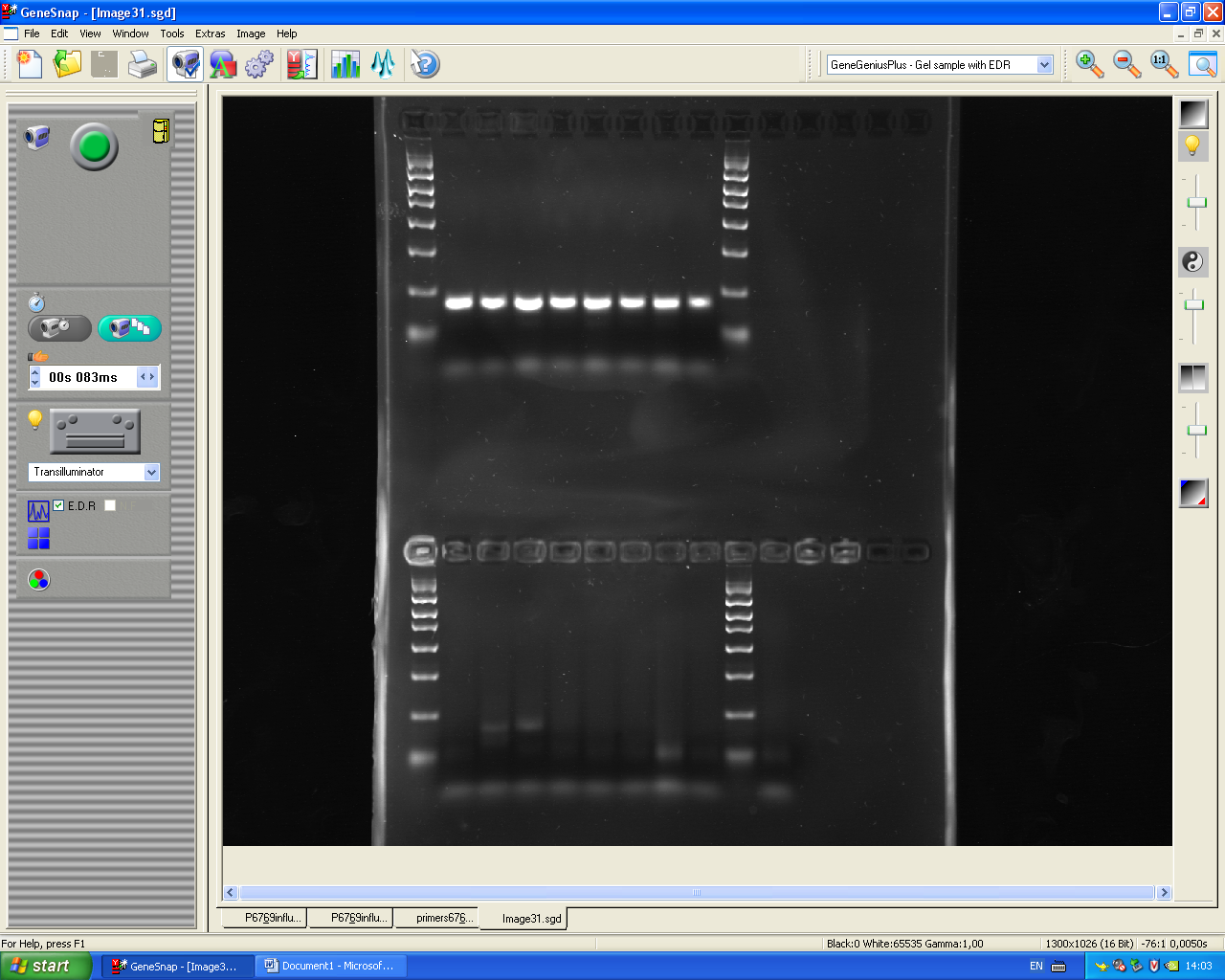 | 5. DLP - GRAM  A B C D E F G H  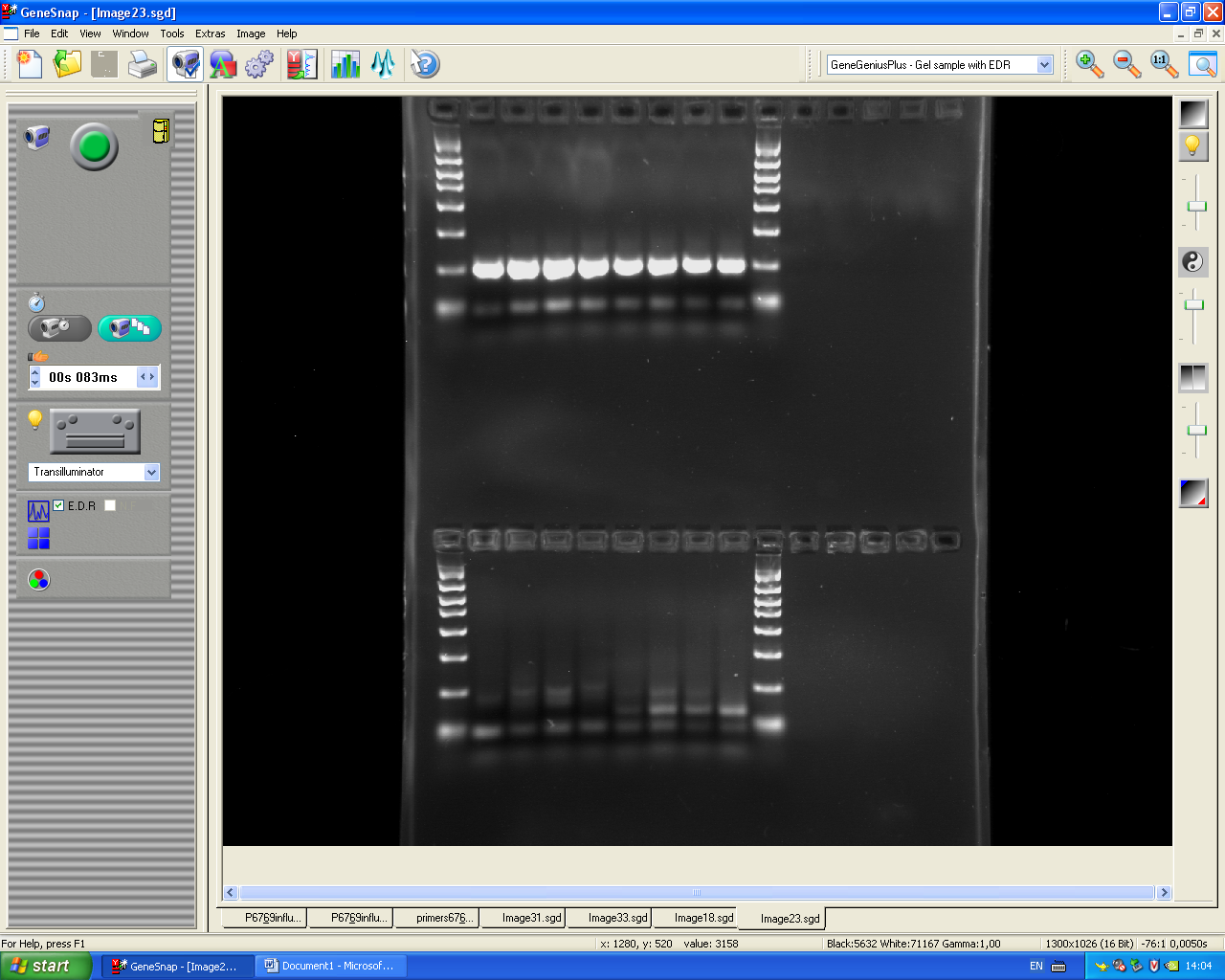 | 6. Zip nucleic acid (ZNA) primers  A B C D E F G H  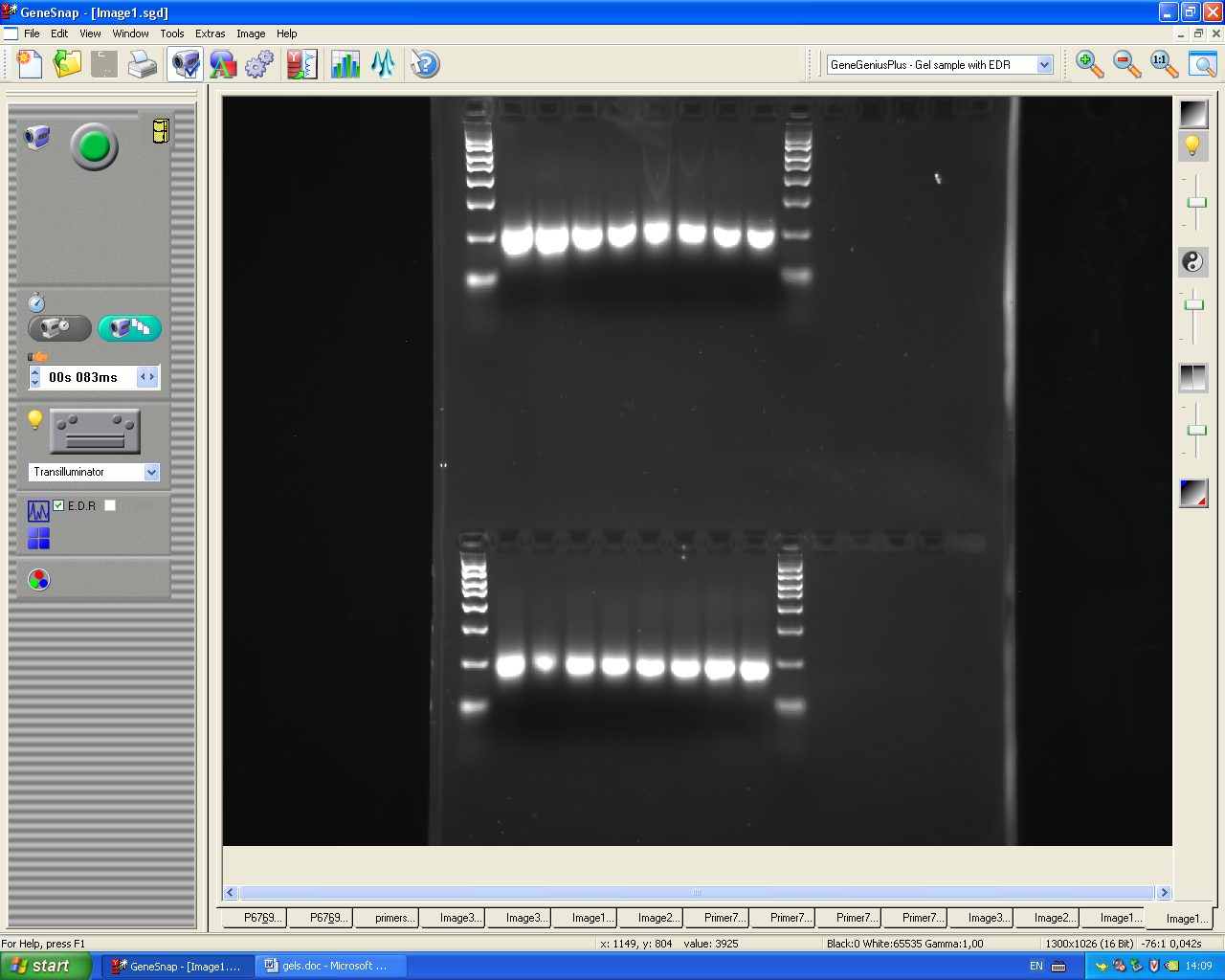 |

**Figure S2:** Agarose gel images of RT-PCR products obtained with 6 different assays using annealing temperature gradient. Letters A – H correspond to the different annealing temperatures tested: A: 65.0 °C, B: 64.1 °C, C: 62.1°C, D: 59.3 °C, E: 55.9 °C, F: 53.0 °C, G: 51.0 °C, H: 50.0 °C. At the extreme wells, a DNA size marker (Sigma) is loaded.

**
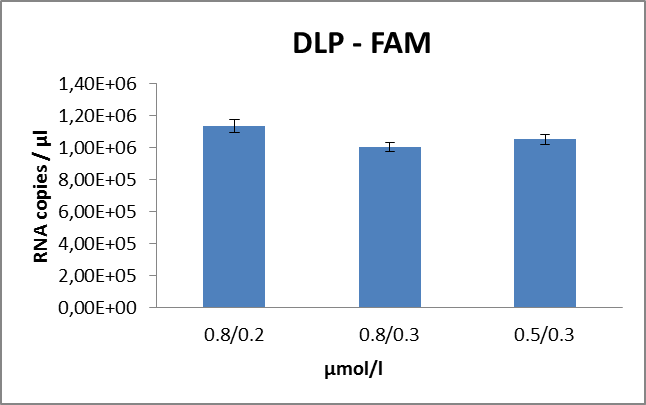

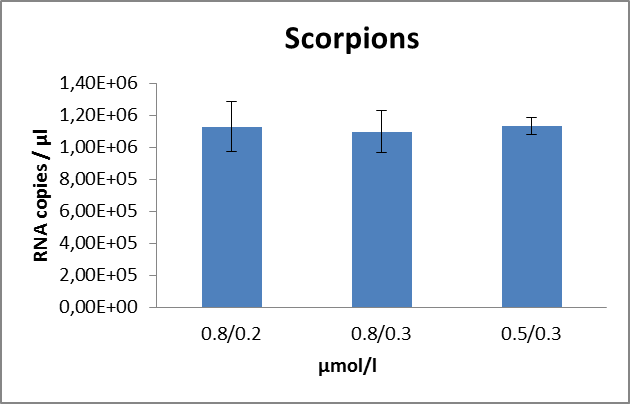

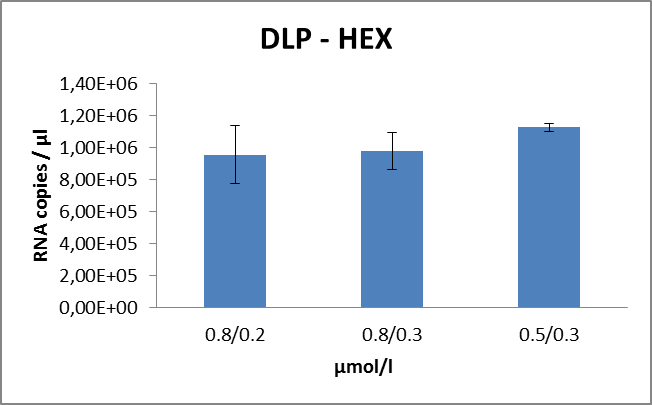
**

**
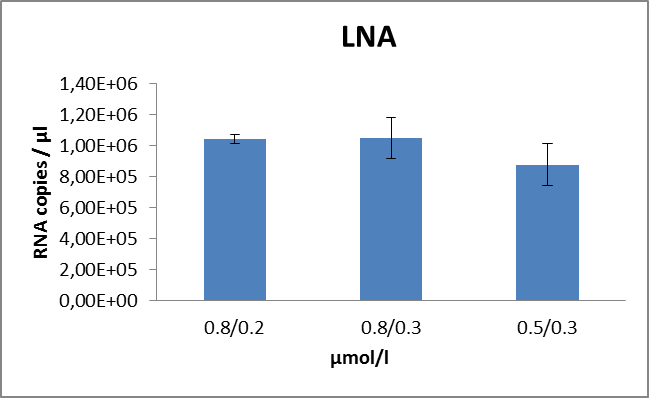

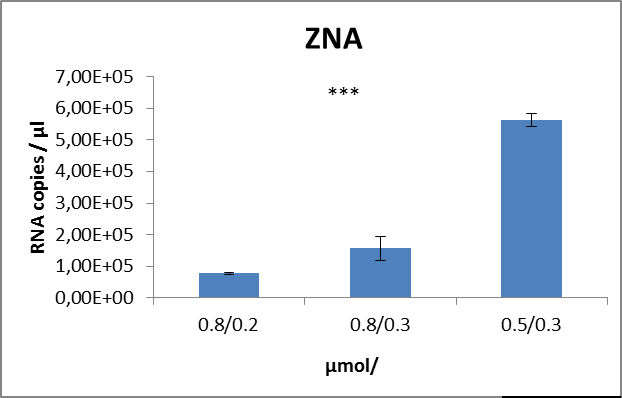

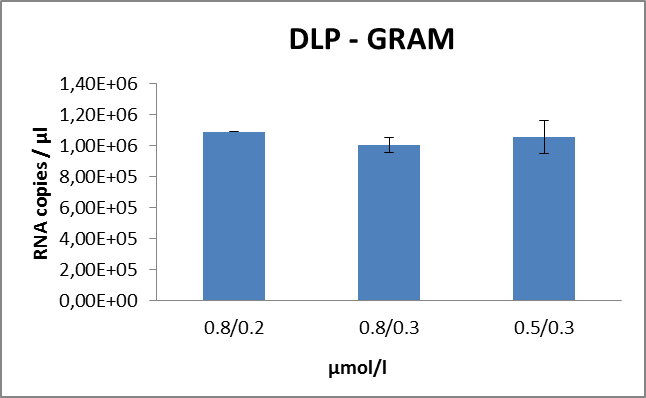
**

**
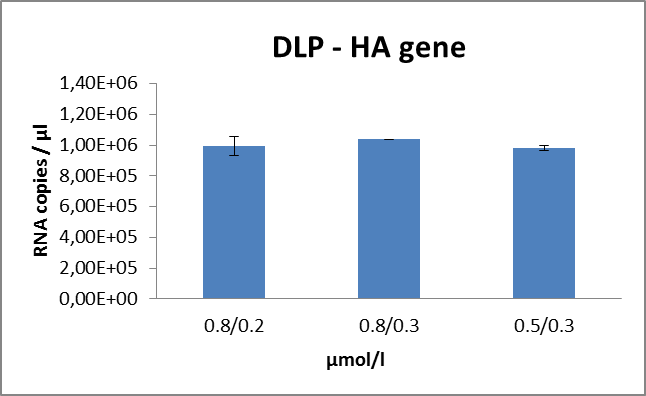
**

**Figure S3**: Optimisation of primer and probe concentration for RNA quantification by RT-dPCR using different methods. Three combinations of primer : probe concentrations were evaluated: i) 0.8 µmol/l : 0.2 µmol/l (ii) 0.8 µmol/l : 0.3 µmol/l and (iii) 0.5 µmol/l : 0.3 µmol/l for 7 methods targeting the influenza A M gene or the HA gene. Each combination is tested in duplicate and error bars indicate the standard deviation (SD). ANOVA single factor analysis revealed significant differences in RNA copy number/microliter for the ZNA method (***).

**SUPPLEMENTAL TABLES**

**Table S1:** Summary of the RT-dPCR measurements (copies/µl) described for IVT RNA (A) and extracted total genomic RNA (B). SD: standard deviation, RSD: relative standard deviation, *u*: combined uncertainty.

A. *In vitro* transcribed RNA

| Primer & probe chemistry | Day | Replicate 1 | Replicate 2 | Replicate 3 | Replicate 4 | Replicate 5 | 1 day mean cp/µl | SD  cp/µl | RSD [%] | 3 day mean cp/µl | Mean RSD [%] | *u*  cp/µl | *u* [%] |
| --- | --- | --- | --- | --- | --- | --- | --- | --- | --- | --- | --- | --- | --- |
| DLP - FAM | 1 | 1.41E+10 | 1.28E+10 | 1.22E+10 | 1.18E+10 | 1.22E+10 | 1.26E+10 | 8.96E+08 | 7.1 |  |  |  |  |
| (FAM-BHQ1) | 2 | 1.02E+10 | 9.66E+09 | 1.06E+10 | 1.08E+10 | 1.03E+10 | 1.03E+10 | 4.32E+08 | 4.2 | 1.16E+10 | 5.6 | 7.36E+08 | 6.3 |
|  | 3 | 1.16E+10 | 1.23E+10 | 1.25E+10 | 1.10E+10 | 1.23E+10 | 1.19E+10 | 6.65E+08 | 5.6 |  |  |  |  |
| DLP - HEX | 1 | 1.24E+10 | 1.06E+10 | 1.18E+10 | 1.22E+10 | 1.22E+10 | 1.18E+10 | 7.20E+08 | 6.1 |  |  |  |  |
| (HEX-BHQ1) | 2 | 1.11E+10 | 1.16E+10 | 1.17E+10 | 1.12E+10 | 1.08E+10 | 1.13E+10 | 3.54E+08 | 3.1 | 1.14E+10 | 5.2 | 3.16E+08 | 2.8 |
|  | 3 | 1.21E+10 | 1.11E+10 | 1.14E+10 | 1.03E+10 | 1.06E+10 | 1.11E+10 | 7.12E+08 | 6.4 |  |  |  |  |
| Scorpions® | 1 | 1.63E+10 | 1.61E+10 | 1.59E+10 | 1.55E+10 | 1.68E+10 | 1.61E+10 | 5.04E+08 | 3.1 |  |  |  |  |
| (FAM-BHQ1) | 2 | 1.59E+10 | 1.62E+10 | 1.55E+10 | 1.53E+10 | 1.61E+10 | 1.58E+10 | 3.75E+08 | 2.4 | 1.57E+10 | 2.8 | 3.34E+08 | 2.1 |
|  | 3 | 1.53E+10 | 1.55E+10 | 1.44E+10 | 1.53E+10 | 1.51E+10 | 1.51E+10 | 4.24E+08 | 2.8 |  |  |  |  |
| Locked nucleic acid (LNA) | 1 | 1.62E+10 | 1.79E+10 | 1.68E+10 | 1.78E+10 | 1.67E+10 | 1.71E+10 | 7.36E+08 | 4.3 |  |  |  |  |
| (FAM-BHQ1) | 2 | 2.02E+10 | 1.80E+10 | 1.77E+10 | 1.70E+10 | 1.92E+10 | 1.84E+10 | 1.27E+09 | 6.9 | 1.77E+10 | 5.7 | 5.45E+08 | 3.1 |
|  | 3 | 1.87E+10 | 1.62E+10 | 1.81E+10 | 1.70E+10 | 1.81E+10 | 1.76E+10 | 1.02E+09 | 5.8 |  |  |  |  |
| Zip nucleic acid (ZNA) | 1 | 1.57E+10 | 1.78E+10 | 1.84E+10 | 1.75E+10 | 1.70E+10 | 1.73E+10 | 1.00E+09 | 5.8 |  |  |  |  |
| (FAM-BHQ1) | 2 | 1.83E+10 | 1.83E+10 | 1.89E+10 | 1.68E+10 | 1.68E+10 | 1.78E+10 | 9.46E+08 | 5.3 | 1.72E+10 | 4.8 | 5.05E+08 | 2.9 |
|  | 3 | 1.72E+10 | 1.62E+10 | 1.66E+10 | 1.64E+10 | 1.58E+10 | 1.64E+10 | 5.47E+08 | 3.3 |  |  |  |  |
| DLP - GRAM | 1 | 1.51E+10 | 1.42E+10 | 1.63E+10 | 1.55E+10 | 1.56E+10 | 1.53E+10 | 7.64E+08 | 5.0 |  |  |  |  |
| (FAM-BHQ1) | 2 | 1.44E+10 | 1.58E+10 | 1.53E+10 | 1.59E+10 | 1.59E+10 | 1.55E+10 | 6.37E+08 | 4.1 | 1.58E+10 | 4.8 | 4.73E+08 | 3.0 |
|  | 3 | 1.54E+10 | 1.60E+10 | 1.77E+10 | 1.64E+10 | 1.70E+10 | 1.65E+10 | 8.89E+08 | 5.4 |  |  |  |  |

B. Extracted total genomic RNA

| Primer & probe chemistry | Target | Day | Replicate 1 | Replicate 2 | Replicate 3 | 1 day mean cp/µl | SD  cp/µl | RSD [%] | 3 day mean cp/µl | Mean RSD [%] | *u*  cp/µl | *u* [%] |
| --- | --- | --- | --- | --- | --- | --- | --- | --- | --- | --- | --- | --- |
| DLP - FAM |  | 1 | 1.00E+06 | 1.06E+06 | 1.10E+06 | 1.06E+06 | 4.80E+04 | 4.5 |  |  |  |  |
| (FAM-BHQ1) | M gene | 2 | 1.04E+06 | 1.03E+06 | 1.03E+06 | 1.03E+06 | 2.13E+03 | 0.2 | 1.09E+06 | 3.8 | 5.09E+04 | 4.7 |
|  |  | 3 | 1.19E+06 | 1.09E+06 | 1.25E+06 | 1.18E+06 | 7.74E+04 | 6.6 |  |  |  |  |
| DLP - HEX |  | 1 | 1.08E+06 | 1.01E+06 | 1.03E+06 | 1.04E+06 | 3.59E+04 | 3.4 |  |  |  |  |
| (HEX-BHQ1) | M gene | 2 | 1.09E+06 | 9.94E+05 | 1.08E+06 | 1.05E+06 | 5.23E+04 | 5.0 | 1.10E+06 | 4.0 | 6.02E+04 | 5.5 |
|  |  | 3 | 1.27E+06 | 1.20E+06 | 1.19E+06 | 1.22E+06 | 4.53E+04 | 3.7 |  |  |  |  |
| DLP – HA gene duplex |  | 1 | 1.08E+06 | 1.04E+06 | 1.04E+06 | 1.06E+06 | 2.21E+04 | 2.1 |  |  |  |  |
| (FAM-BHQ1) | HA gene (H3) | 2 | 9.66E+05 | 1.02E+06 | 1.02E+06 | 1.00E+06 | 2.97E+04 | 3.0 | 1.06E+06 | 4.0 | 4.53E+04 | 4.3 |
|  | (duplex) | 3 | 1.20E+06 | 1.05E+06 | 1.15E+06 | 1.13E+06 | 7.76E+04 | 6.8 |  |  |  |  |
| DLP - HEX duplex |  | 1 | 1.06E+06 | 1.01E+06 | 9.78E+05 | 1.02E+06 | 4.08E+04 | 4.0 |  |  |  |  |
| (HEX-BHQ1) | M gene | 2 | 1.01E+06 | 1.04E+06 | 1.04E+06 | 1.03E+06 | 1.73E+04 | 1.7 | 1.08E+06 | 2.9 | 5.88E+04 | 5.4 |
|  | (duplex) | 3 | 1.17E+06 | 1.23E+06 | 1.18E+06 | 1.19E+06 | 3.53E+04 | 3.0 |  |  |  |  |
| Scorpions® |  | 1 | 1.24E+06 | 1.10E+06 | 1.05E+06 | 1.13E+06 | 1.01E+05 | 8.9 |  |  |  |  |
| (FAM-BHQ1) | M gene | 2 | 1.21E+06 | 1.19E+06 | 1.08E+06 | 1.16E+06 | 7.26E+04 | 6.2 | 1.19E+06 | 6.8 | 5.95E+04 | 5.0 |
|  |  | 3 | 1.28E+06 | 1.35E+06 | 1.22E+06 | 1.28E+06 | 6.57E+04 | 5.1 |  |  |  |  |
| Locked nucleic acid (LNA) |  | 1 | 9.86E+05 | 1.12E+06 | 9.82E+05 | 1.03E+06 | 8.01E+04 | 7.8 |  |  |  |  |
| (FAM-BHQ1) | M gene | 2 | 1.02E+06 | 1.07E+06 | 1.04E+06 | 1.04E+06 | 3.00E+04 | 2.9 | 1.09E+06 | 4.2 | 5.50E+04 | 5.1 |
|  |  | 3 | 1.21E+06 | 1.16E+06 | 1.18E+06 | 1.18E+06 | 2.31E+04 | 1.9 |  |  |  |  |
| Zip nucleic acid (ZNA) |  | 1 | 1.35E+06 | 1.24E+06 | 1.15E+06 | 1.25E+06 | 1.02E+05 | 8.2 |  |  |  |  |
| (HEX-BHQ1) | M gene | 2 | 1.14E+06 | 1.14E+06 | 1.25E+06 | 1.17E+06 | 6.32E+04 | 5.4 | 1.27E+06 | 5.1 | 7.22E+04 | 5.7 |
|  |  | 3 | 1.38E+06 | 1.42E+06 | 1.38E+06 | 1.39E+06 | 2.59E+04 | 1.9 |  |  |  |  |
| DLP -GRAM |  | 1 | 1.17E+06 | 1.08E+06 | 1.07E+06 | 1.10E+06 | 5.46E+04 | 4.9 |  |  |  |  |
| (FAM-BHQ1) | M gene | 2 | 1.14E+06 | 1.14E+06 | 1.25E+06 | 1.17E+06 | 6.32E+04 | 5.4 | 1.14E+06 | 4.8 | 3.33E+04 | 2.9 |
|  |  | 3 | 1.19E+06 | 1.10E+06 | 1.17E+06 | 1.16E+06 | 4.75E+04 | 4.1 |  |  |  |  |
| DLP- HA gene |  | 1 | 1.12E+06 | 1.08E+06 | 1.06E+06 | 1.09E+06 | 3.37E+04 | 3.1 |  |  |  |  |
| (FAM-BHQ1) | HA gene (H3) | 2 | 1.01E+06 | 1.04E+06 | 1.04E+06 | 1.03E+06 | 1.73E+04 | 1.7 | 1.13E+06 | 2.7 | 7.72E+04 | 6.8 |
|  |  | 3 | 1.23E+06 | 1.32E+06 | 1.29E+06 | 1.28E+06 | 4.33E+04 | 3.4 |  |  |  |  |

**Table S2:** Single-factor analysis of variance (ANOVA) results for IVT RNA (A) and extracted total genomic RNA (B).

**A.** *In vitro* transcribed RNA

| ANOVA |  |  |  |  |  |  |
| --- | --- | --- | --- | --- | --- | --- |
| *Source of Variation* | *SS* | *df* | *MS* | *F* | *P-value* | *F crit* |
| Between Groups | 5.60648E+20 | 5 | 1.1213E+20 | 128.3069294 | 8.40E-38 | 2.323126498 |
| Within Groups | 7.3409E+19 | 84 | 8.73916E+17 |  |  |  |
|  |  |  |  |  |  |  |
| Total | 6.34057E+20 | 89 |  |  |  |  |

B. Extracted total genomic RNA

| ANOVA |  |  |  |  |  |  |
| --- | --- | --- | --- | --- | --- | --- |
| *Source of Variation* | *SS* | *df* | *MS* | *F* | *P-value* | *F crit* |
| Between Groups | 3.15349E+11 | 8 | 39418652099 | 4.700386046 | 0.00011648 | 2.069831642 |
| Within Groups | 6.03811E+11 | 72 | 8386258429 |  |  |  |
|  |  |  |  |  |  |  |
| Total | 9.1916E+11 | 80 |  |  |  |  |

**Table S3:** Tukey's HSD test results for IVT RNA (A) and extracted total genomic RNA (B). *n*1: number of replicates of method 1, *n*2: number of replicates of method 2, critical *q*: critical value for the Tukey HSD test, with α, the type I error rate (0.05), r, the number of groups and df_w_, the degrees of freedom of *MS*within of ANOVA results, 95 % CI: the 95 % confidence interval.

Q statistics value* is calculated as $\text{q}=\frac{\left| \bar{x}_{i}- \bar{x}_{j} \right|}{\sqrt{{{MS}_{\mathrm{within}}}/n}}$ . Differences between measurement results of 2 methods are significant if the q statistics value > the critical *q* value and they are indicated in bold/italic in the table.

**A.** *In vitro* transcribed RNA

| Method 1 | Method 2 | *n*1 | *n*2 | Mean 1 | Mean 2 | Mean 1 - Mean 2 | Critical *q* (α, r, df_w_) | *q* value* | 95 % CI | |
| --- | --- | --- | --- | --- | --- | --- | --- | --- | --- | --- |
| DLP- FAM | DLP - HEX | 15 | 15 | 1.16E+10 | 1.14E+10 | 2.29E+08 | 4.13 | 0.9 | 2.29E+08 | 2.29E+08 |
|  | Scorpions | 15 | 15 | 1.16E+10 | 1.57E+10 | 4.05E+09 | 4.13 | ***16.8*** | 4.05E+09 | 4.05E+09 |
|  | LNA | 15 | 15 | 1.16E+10 | 1.77E+10 | 6.07E+09 | 4.13 | ***25.2*** | 6.07E+09 | 6.07E+09 |
|  | ZNA | 15 | 15 | 1.16E+10 | 1.72E+10 | 5.54E+09 | 4.13 | ***23.0*** | 5.54E+09 | 5.54E+09 |
|  | DLP - GRAM | 15 | 15 | 1.16E+10 | 1.58E+10 | 4.15E+09 | 4.13 | ***17.2*** | 4.15E+09 | 4.15E+09 |
| DLP - HEX | Scorpions | 15 | 15 | 1.14E+10 | 1.57E+10 | 4.28E+09 | 4.13 | ***17.7*** | 4.28E+09 | 4.28E+09 |
|  | LNA | 15 | 15 | 1.14E+10 | 1.77E+10 | 6.30E+09 | 4.13 | ***26.1*** | 6.30E+09 | 6.30E+09 |
|  | ZNA | 15 | 15 | 1.14E+10 | 1.72E+10 | 5.77E+09 | 4.13 | ***23.9*** | 5.77E+09 | 5.77E+09 |
|  | DLP - GRAM | 15 | 15 | 1.14E+10 | 1.58E+10 | 4.38E+09 | 4.13 | ***18.2*** | 4.38E+09 | 4.38E+09 |
| Scorpions | LNA | 15 | 15 | 1.57E+10 | 1.77E+10 | 2.02E+09 | 4.13 | ***8.4*** | 2.02E+09 | 2.02E+09 |
|  | ZNA | 15 | 15 | 1.57E+10 | 1.72E+10 | 1.49E+09 | 4.13 | ***6.2*** | 1.49E+09 | 1.49E+09 |
|  | DLP - GRAM | 15 | 15 | 1.57E+10 | 1.58E+10 | 1.00E+08 | 4.13 | 0.4 | 1.00E+08 | 1.00E+08 |
| LNA | ZNA | 15 | 15 | 1.77E+10 | 1.72E+10 | 5.31E+08 | 4.13 | 2.2 | 5.31E+08 | 5.31E+08 |
|  | DLP - GRAM | 15 | 15 | 1.77E+10 | 1.58E+10 | 1.92E+09 | 4.13 | ***8.0*** | 1.92E+09 | 1.92E+09 |
| ZNA | DLP - GRAM | 15 | 15 | 1.72E+10 | 1.58E+10 | 1.39E+09 | 4.13 | ***5.8*** | 1.39E+09 | 1.39E+09 |

B. Extracted total genomic RNA

| Method 1 | Method 2 | *n*1 | *n*2 | Mean 1 | Mean 2 | Mean 1 - Mean 2 | Critical *q*(α, r, dfw) | *q* value * | 95 % CI | |
| --- | --- | --- | --- | --- | --- | --- | --- | --- | --- | --- |
| DLP- FAM | DLP - HEX | 9 | 9 | 1.09E+06 | 1.10E+06 | 1.54E+04 | 4.54 | 0.50 | 1.54E+04 | 1.54E+04 |
|  | DLP - HA gene_Duplex | 9 | 9 | 1.09E+06 | 1.06E+06 | 2.54E+04 | 4.54 | 0.83 | 2.54E+04 | 2.54E+04 |
|  | DLP - HEX_Duplex | 9 | 9 | 1.09E+06 | 1.08E+06 | 8.15E+03 | 4.54 | 0.27 | 8.15E+03 | 8.15E+03 |
|  | Scorpions | 9 | 9 | 1.09E+06 | 1.19E+06 | 1.02E+05 | 4.54 | 3.35 | 1.02E+05 | 1.02E+05 |
|  | LNA | 9 | 9 | 1.09E+06 | 1.09E+06 | 3.63E+03 | 4.54 | 0.12 | 3.63E+03 | 3.63E+03 |
|  | ZNA | 9 | 9 | 1.09E+06 | 1.27E+06 | 1.82E+05 | 4.54 | ***5.97*** | 1.82E+05 | 1.82E+05 |
|  | DLP - GRAM | 9 | 9 | 1.09E+06 | 1.14E+06 | 5.55E+04 | 4.54 | 1.82 | 5.55E+04 | 5.56E+04 |
|  | DLP - HA gene | 9 | 9 | 1.09E+06 | 1.13E+06 | 4.33E+04 | 4.54 | 1.42 | 4.33E+04 | 4.33E+04 |
| DLP - HEX | DLP - HA gene_Duplex | 9 | 9 | 1.10E+06 | 1.06E+06 | 4.08E+04 | 4.54 | 1.34 | 4.08E+04 | 4.08E+04 |
|  | DLP - HEX_Duplex | 9 | 9 | 1.10E+06 | 1.08E+06 | 2.35E+04 | 4.54 | 0.77 | 2.35E+04 | 2.36E+04 |
|  | Scorpions | 9 | 9 | 1.10E+06 | 1.19E+06 | 8.69E+04 | 4.54 | 2.85 | 8.69E+04 | 8.69E+04 |
|  | LNA | 9 | 9 | 1.10E+06 | 1.09E+06 | 1.90E+04 | 4.54 | 0.62 | 1.90E+04 | 1.90E+04 |
|  | ZNA | 9 | 9 | 1.10E+06 | 1.27E+06 | 1.67E+05 | 4.54 | ***5.46*** | 1.67E+05 | 1.67E+05 |
|  | DLP - GRAM | 9 | 9 | 1.10E+06 | 1.14E+06 | 4.01E+04 | 4.54 | 1.32 | 4.01E+04 | 4.02E+04 |
|  | DLP - HA gene | 9 | 9 | 1.10E+06 | 1.13E+06 | 2.79E+04 | 4.54 | 0.91 | 2.79E+04 | 2.79E+04 |
| DLP - HEX_Duplex | DLP - HEX_Duplex | 9 | 9 | 1.06E+06 | 1.08E+06 | 1.73E+04 | 4.54 | 0.57 | 1.73E+04 | 1.73E+04 |
|  | Scorpions | 9 | 9 | 1.06E+06 | 1.19E+06 | 1.28E+05 | 4.54 | 4.18 | 1.28E+05 | 1.28E+05 |
|  | LNA | 9 | 9 | 1.06E+06 | 1.09E+06 | 2.18E+04 | 4.54 | 0.71 | 2.18E+04 | 2.18E+04 |
|  | ZNA | 9 | 9 | 1.06E+06 | 1.27E+06 | 2.08E+05 | 4.54 | ***6.80*** | 2.08E+05 | 2.08E+05 |
|  | DLP - GRAM | 9 | 9 | 1.06E+06 | 1.14E+06 | 8.10E+04 | 4.54 | 2.65 | 8.09E+04 | 8.10E+04 |
|  | DLP - HA gene | 9 | 9 | 1.06E+06 | 1.13E+06 | 6.87E+04 | 4.54 | 2.25 | 6.87E+04 | 6.87E+04 |
| DLP - HEX_Duplex | Scorpions | 9 | 9 | 1.08E+06 | 1.19E+06 | 1.10E+05 | 4.54 | 3.62 | 1.10E+05 | 1.10E+05 |
|  | LNA | 9 | 9 | 1.08E+06 | 1.09E+06 | 4.52E+03 | 4.54 | 0.15 | 4.52E+03 | 4.52E+03 |
|  | ZNA | 9 | 9 | 1.08E+06 | 1.27E+06 | 1.90E+05 | 4.54 | ***6.23*** | 1.90E+05 | 1.90E+05 |
|  | DLP - GRAM | 9 | 9 | 1.08E+06 | 1.14E+06 | 6.37E+04 | 4.54 | 2.09 | 6.37E+04 | 6.37E+04 |
|  | DLP - HA gene | 9 | 9 | 1.08E+06 | 1.13E+06 | 5.14E+04 | 4.54 | 1.68 | 5.14E+04 | 5.14E+04 |
| Scorpions | LNA | 9 | 9 | 1.19E+06 | 1.09E+06 | 1.06E+05 | 4.54 | 3.47 | 1.06E+05 | 1.06E+05 |
|  | ZNA | 9 | 9 | 1.19E+06 | 1.27E+06 | 7.98E+04 | 4.54 | 2.61 | 7.98E+04 | 7.98E+04 |
|  | DLP - GRAM | 9 | 9 | 1.19E+06 | 1.14E+06 | 4.68E+04 | 4.54 | 1.53 | 4.68E+04 | 4.68E+04 |
|  | DLP - HA gene | 9 | 9 | 1.19E+06 | 1.13E+06 | 5.90E+04 | 4.54 | 1.93 | 5.90E+04 | 5.90E+04 |
| LNA | ZNA | 9 | 9 | 1.09E+06 | 1.27E+06 | 1.86E+05 | 4.54 | ***6.09*** | 1.86E+05 | 1.86E+05 |
|  | DLP - GRAM | 9 | 9 | 1.09E+06 | 1.14E+06 | 5.92E+04 | 4.54 | 1.94 | 5.92E+04 | 5.92E+04 |
|  | DLP - HA gene | 9 | 9 | 1.09E+06 | 1.13E+06 | 4.69E+04 | 4.54 | 1.54 | 4.69E+04 | 4.69E+04 |
| ZNA | DLP - GRAM | 9 | 9 | 1.27E+06 | 1.14E+06 | 1.27E+05 | 4.54 | 4.15 | 1.27E+05 | 1.27E+05 |
|  | DLP - HA gene | 9 | 9 | 1.27E+06 | 1.13E+06 | 1.39E+05 | 4.54 | ***4.55*** | 1.39E+05 | 1.39E+05 |
| DLP - GRAM | DLP - HA gene | 9 | 9 | 1.14E+06 | 1.13E+06 | 1.23E+04 | 4.54 | 0.40 | 1.23E+04 | 1.23E+04 |
